# Supplementary material for: Ceramic-carbon Janus membrane for robust solar-thermal desalination
Source: Nat Commun. 2025 Mar 18;16:2659. doi: 10.1038/s41467-025-57888-2 (PMC11920389; doi:10.1038/s41467-025-57888-2)
Supplement: Supplementary file 1 — Supplementary Information [file 41467_2025_57888_MOESM1_ESM.pdf]

Supplementary Information

Ceramic-Carbon Janus Membrane for Robust Solar-Thermal Desalination

Yingchao Dong[1], Camille Violet[2], Chunyi Sun[3], Xianhui Li[4], Yuxuan Sun[1], Qingbin Zheng[1*], Chuyang Tang[5*], Menachem Elimelech[6,7*]*

[1] School of Science and Engineering, The Chinese University of Hong Kong, Shenzhen, Guangdong Province, 518172, China

[2] Department of Chemical and Environmental Engineering, Yale University, New Haven, CT 06520-8286, USA

[3] School of Water Conservancy and Environment, Jinan University, Jinan 250022, China

[4] Guangdong Provincial Key Laboratory of Water Quality Improvement and Ecological Restoration for Watersheds, School of Ecology, Environment and Resources, Guangdong University of Technology, Guangzhou, 510006, China

[5] Department of Civil Engineering, The University of Hong Kong, Pokfulam, Hong Kong, China

[6] Department of Civil and Environmental Engineering, Rice University, Houston, TX, 77005, USA

[7] Department of Chemical and Biomolecular Engineering, Rice University, Houston, TX, 77005, USA

Number of pages (including the cover page): 43

Number of figures: 29

Number of tables: 6

1. Supplementary Methods: Membrane Fabrication and Characterization (Supplementary Page 3)

2. Supplementary Results: Properties of Ceramic-carbon Janus Membranes (Supplementary Page 10)

3. Supplementary Results: Enhanced Solar Absorption and Solar-thermal Effect (Supplementary Page 18)

4. Supplementary Results: Solar-thermal Desalination Performance (Supplementary Page 21)

5. Supplementary Methods: Simulation Details and Results (Supplementary Page 25)

6. Supplementary Results: Solar-thermal Desalination of Challenging Saline Waters (Supplementary Page 33)

7. Supplementary Results: Design and Solar-thermal Desalination Performance of Flat Membranes (Supplementary Page 37)

Supplementary References (Supplementary Page 41)

S1 Supplementary Methods: Membrane Fabrication and Characterization

S1.1 Raw Materials and Chemical Reagents

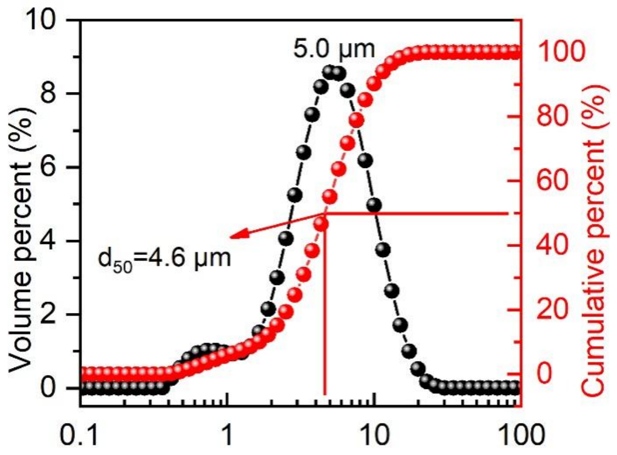

Supplementary Figure 1. Particle size distribution of α-Al_2O_3 powder. The particle size was determined using a laser particle size analyzer (Marvin 2000, Malvern instruments Ltd., UK). The α-Al_2O_3 powder has a particle size of d_{50} = 4.6 μm.

Supplementary Table 1. Materials and chemical reagents.

Material	Molecular formula	Purity	Manufacturer
Alumina powder	α-Al_2O_3	99%	a
N-methyl-2-pyrrolidone (NMP)	C_5H_9NO	AR	b
Polyvinylpyrrolidone (PVP) (MW ~ 30000)	$(C_6H_9NO)_n$	AR	c
Poly(ethersulfone) (PES)	$(C_{12}H_8O_3S)_n$	AR	d
Sodium chloride	NaCl	AR	b
Calcium chloride	$CaCl_2$	AR	b
Sodium sulfate	Na_2SO_4	AR	b
Ethylene	C_2H_4	≥99.999%	e
Hydrogen	H_2	≥99.999%	e
Nitrogen	N_2	≥99.999%	e

Notes: a: Shanghai Jingrui Chemical Co., Ltd., China; b: Damao Chemical Reagent Factory, Tianjin, China; c: Sinopharm Chemical Reagent Co., Ltd., China; d: Bei-Shi-De Synthetic Plastics Company; e: Dalian Guanghui Gas Co., Ltd., China.

S1.2 Fabrication of Ceramic Membrane

Supplementary Table 2. Suspension compositions and dry-wet spinning parameters for preparation of ceramic membrane.

Compositions and spinning parameters	Conditions
Solid state loading (wt.%)	50
PES+NMP (wt.%)	49 (PES: NMP = 1:4)
PVP (wt.%)	1
Temperature (°C)	25
External coagulant	Deionized water
Bore liquid flow rate (mL min^{-1})	20
Nitrogen pressure (MPa)	0.15
Air gap (cm)	15

Notes: PES: polyethersulfone, NMP: N-methyl-2-pyrrolidone, PVP: Polyvinylpyrrolidone.

Briefly, polyethersulfone (PES, 39.2 wt.%) and polyvinylpyrrolidone (PVP, 1 wt.%) were dissolved in N-methyl-2-pyrrolidone (NMP, 9.8 wt.%) with constant stirring for 6 h to form a homogeneous polymer solution. Then, α-Al_2O_3 powder (50 wt.%) was gradually added into the above polymer solution. After ball-milling for 48 h, the suspension was vacuum-degassed for 2 h to remove air bubbles. Next, the suspension was extruded by a laboratory-made tube-in-orifice spinneret (inner diameter: 1.3 mm, outer diameter: 2.5 mm) with a constant N_2 pressure (0.15 MPa). The air gap distance between the coagulant bath and the spinneret was controlled to 15 cm. Deionized (DI) water was pumped through the center bore of the spinneret at a constant flow rate of 20 mL min^{-1}. DI water and tap water were used as the internal and external coagulants, respectively. The Al_2O_3 membrane green bodies were immersed in a water bath for 24 h to complete a phase inversion process. After fully drying in air at room temperature (25 °C), the fabricated precursor hollow fibers were sintered in a muffle furnace for 2 h at 600 °C to remove residual water and the organic polymers (Supplementary Figure 2). They were calcined at a certain temperature (1450–1600 °C) for 4 h in air to get the final Al_2O_3 ceramic membranes. The details of

suspension compositions and dry-wet spinning parameters are shown in Supplementary Table 2.

The three-point bending mechanical strength of aluminum ceramic membranes was tested by using a universal testing machine (AGS-X, Shimadzu Instruments Manufacturing Co., Ltd., Suzhou, China). The membranes were placed on the span (20 mm) and loaded at a constant crosshead speed (0.02 mm min^{-1}) until fracture. Ten times measurements at different locations were performed to obtain an average value. The bending strength (σ_F, MPa) of membranes is expressed as follows:[1]

$$\sigma_F = \frac{8FLD}{\pi(D^4 - d^4)} \quad (1)$$

Where D and d are the outer and inner diameters (mm) of aluminum ceramic membrane, respectively. L is the span (20 mm, in this work), F is the tested force (N) at which fracture occurred.

Nitrogen permeance and pore size distribution of the membranes were tested in a Micro-Filtration Membrane Porometer (PSDA-20, Nanjing GaoQ Functional Materials Co., Ltd., China). Water permeance was measured by a laboratory-made filtration device at an operating pressure of 0.5 bar, where liquid water entry pressure was also measured. Each sample was tested at least three times.

S1.3 Characterization of Ceramic Membrane

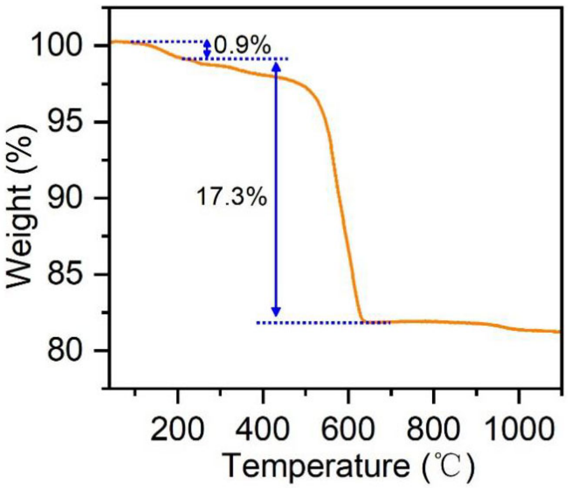

Supplementary Figure 2. Thermogravimetric (TG) curve of the dried aluminum ceramic membrane green body heated between 60 °C and 1100 °C under air atmosphere with a constant heating rate of 10 °C min^{-1}.

S1.4 Structure and Property Regulation of Ceramic Membrane

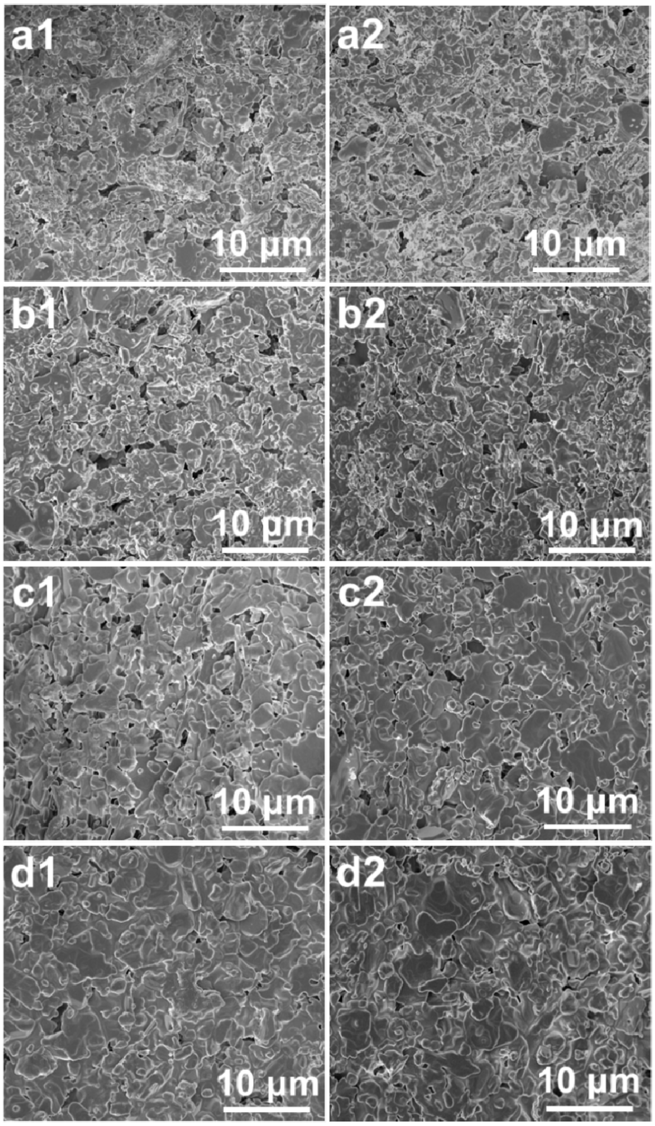

Supplementary Figure 3. SEM images of aluminum ceramic membranes after sintering in air at different temperatures: (a1-a2) 1450 °C, (b1-b2) 1500 °C, (c1-c2) 1550 °C, (d1-d2) 1600 °C. (1: outer surface, 2: inner surfaces).

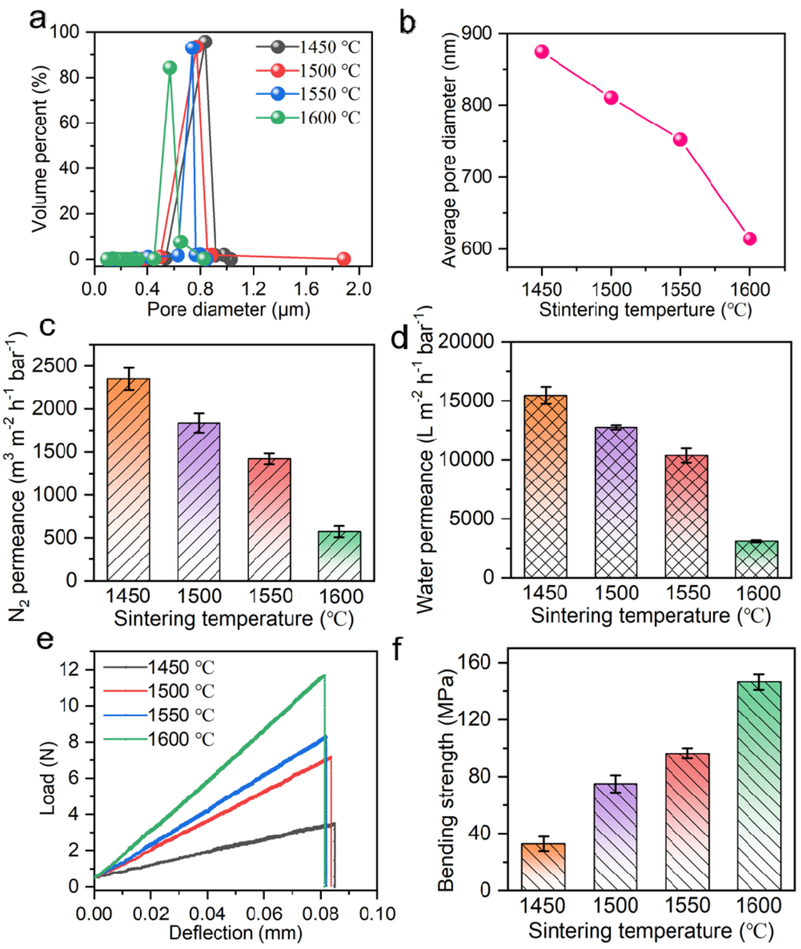

Supplementary Figure 4. Properties of aluminum ceramic membranes sintered at different temperature: (a) pore size distribution, (b) average pore size, (c) N_2 permeance, (d) water permeance, (e) typical load-deflection curves and (f) bending strength.

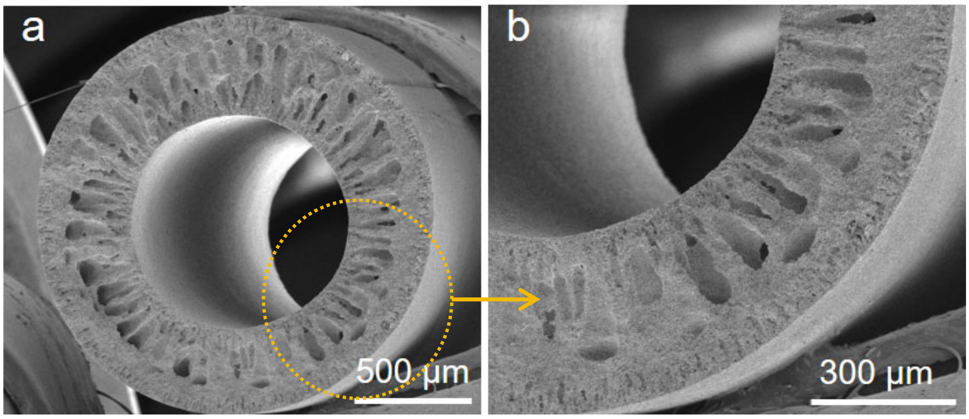

Supplementary Figure 5. SEM images of aluminum ceramic membrane: (a) cross-sectional SEM image and (b) locally enlarged cross-sectional SEM image.

S2 Supplementary Results: Properties of Ceramic-carbon Janus Membranes

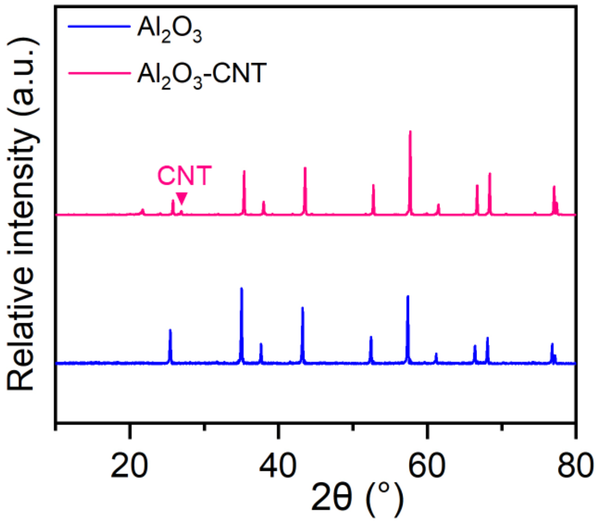

Supplementary Figure 6. XRD patterns of ceramic membrane (Al_2O_3) and ceramic-carbon Janus membrane (Al_2O_3-CNT). The appearance of a reflection at 2θ = 26.5° reveals the formation of CNT layer via chemical vapor deposition.

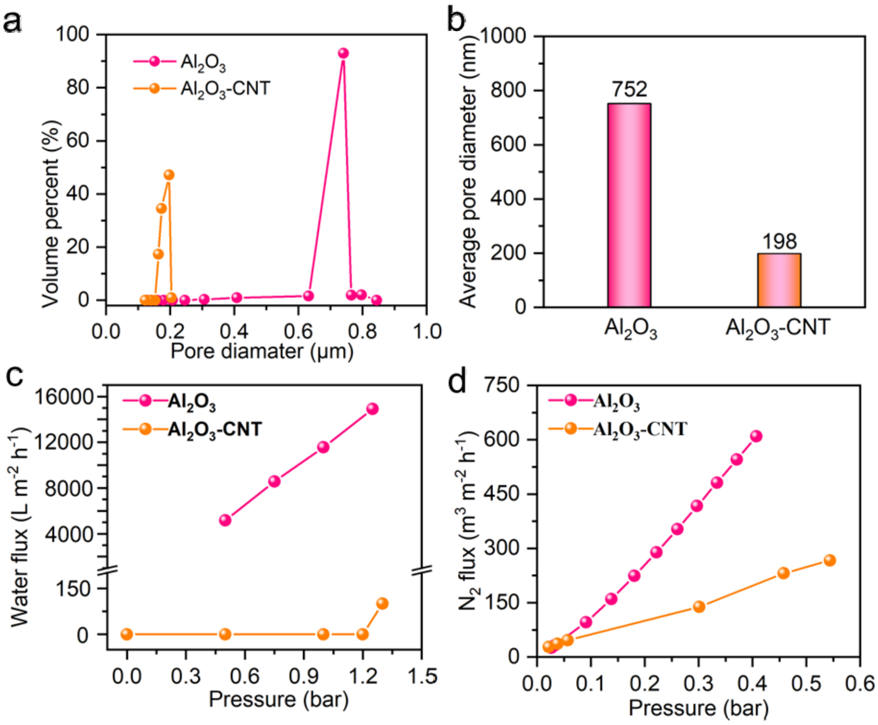

Supplementary Figure 7. Pore size and flux of ceramic membranes (Al_2O_3) and ceramic-carbon Janus membranes (Al_2O_3-CNT): (a) pore size distribution, (b) average pore size, (c) pure water flux and (d) N_2 gas flux. For the ceramic-carbon Janus membranes, the average pore diameter is 198 nm, while the liquid entry pressure (LEP) is 1.2 bar.

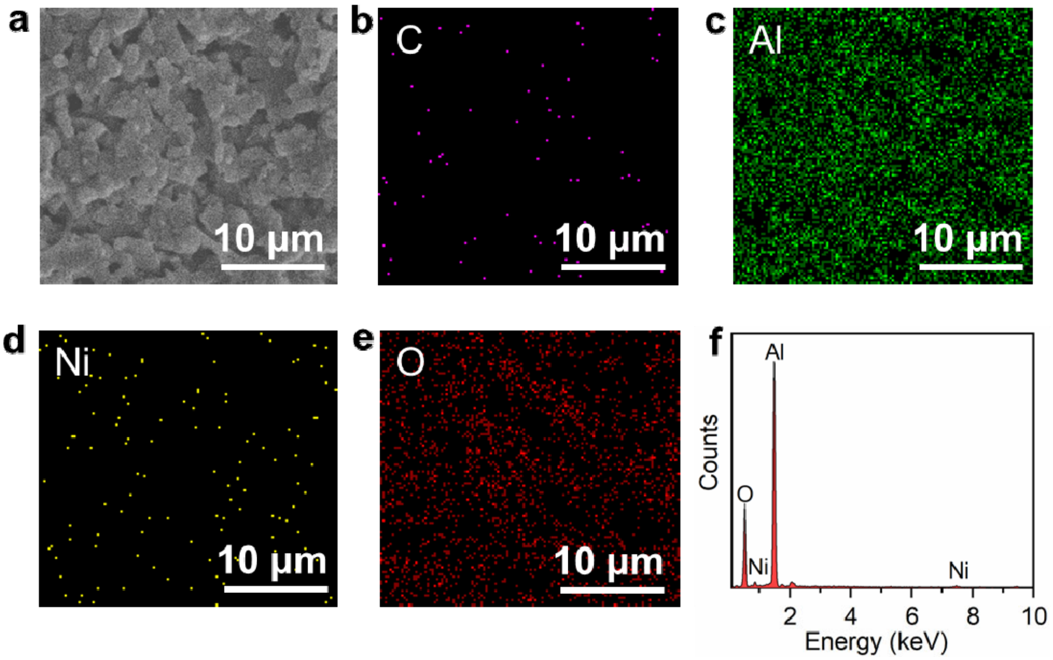

Supplementary Figure 8. SEM and EDS characterization of NiO-loaded aluminum ceramic membrane: (a) surface SEM image, (b-e) EDS images and (f) EDS spectrum (scanned across the whole surface SEM image).

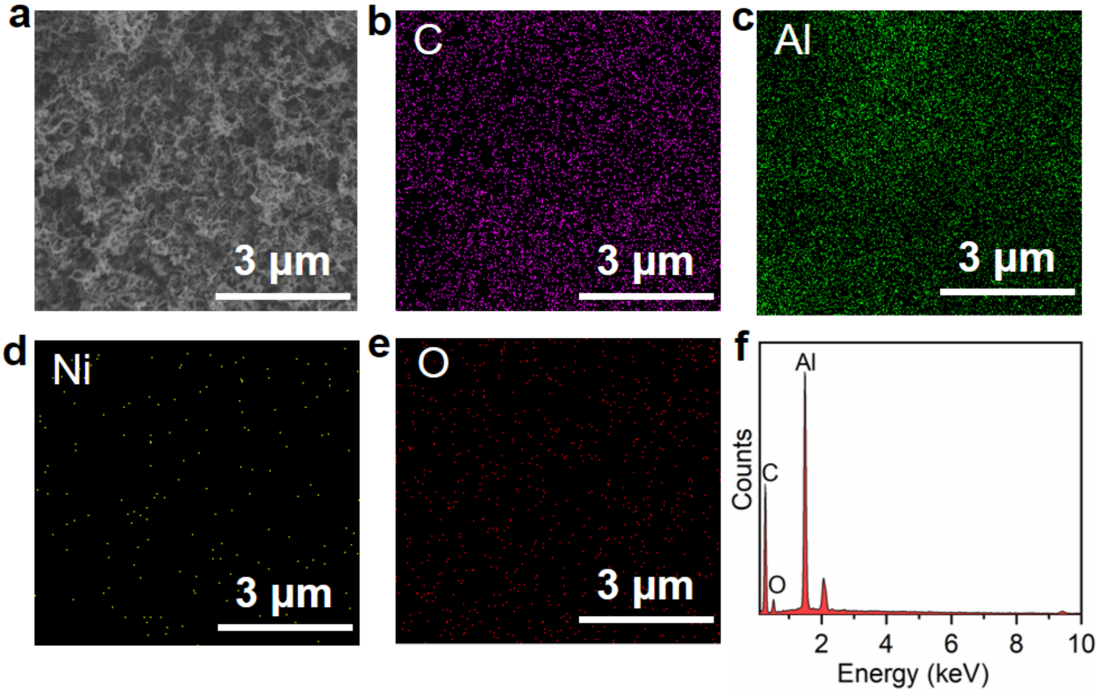

Supplementary Figure 9. SEM and EDS characterization of ceramic-carbon Janus membrane: (a) surface SEM image, (b-e) EDS images and (f) EDS spectrum (scanned across the whole surface SEM image).

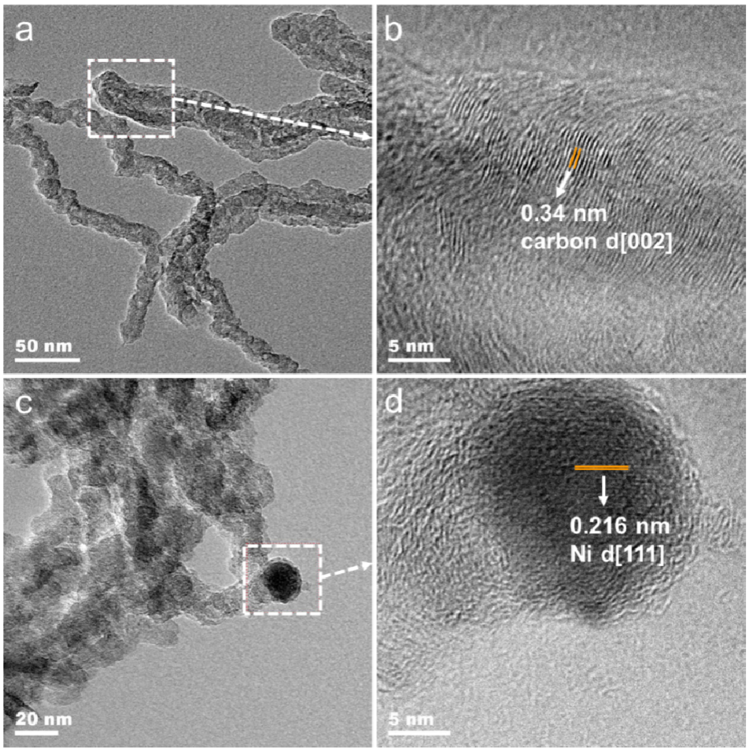

Supplementary Figure 10. Structure and morphology of CNT in ceramic-carbon Janus membrane: (a) TEM and (b) HRTEM image of CNT, (c) TEM image of CNT with Ni particle on the tip and (d) HRTEM image of Ni particle inside the formed CNT.

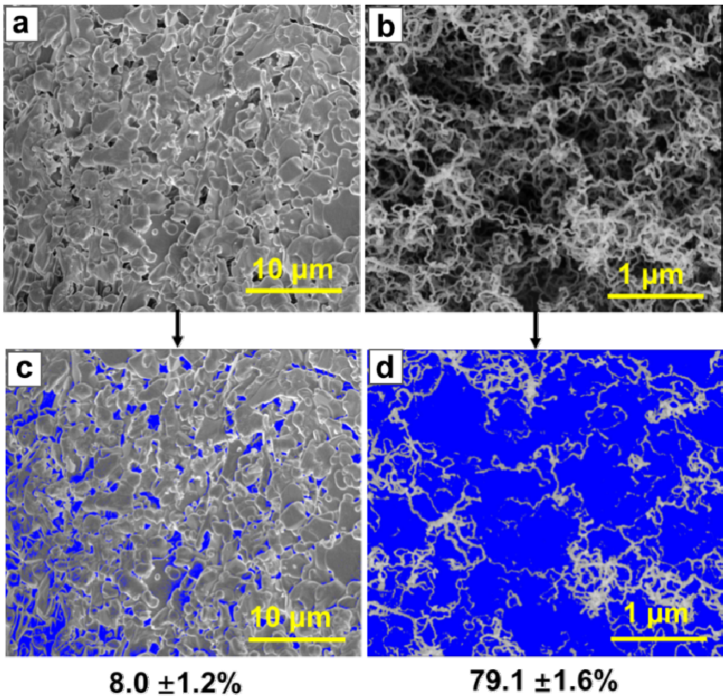

Supplementary Figure 11. Surface SEM images and Image J processed images of ceramic membrane and ceramic-carbon Janus membrane: (a) SEM image of ceramic membrane, (b) SEM image of ceramic-carbon Janus membrane, (c) Image J processed image of ceramic membrane and (d) Image J processed image of ceramic-carbon Janus membrane. The blue area in Figures 11c and 11d denotes surface porosity.

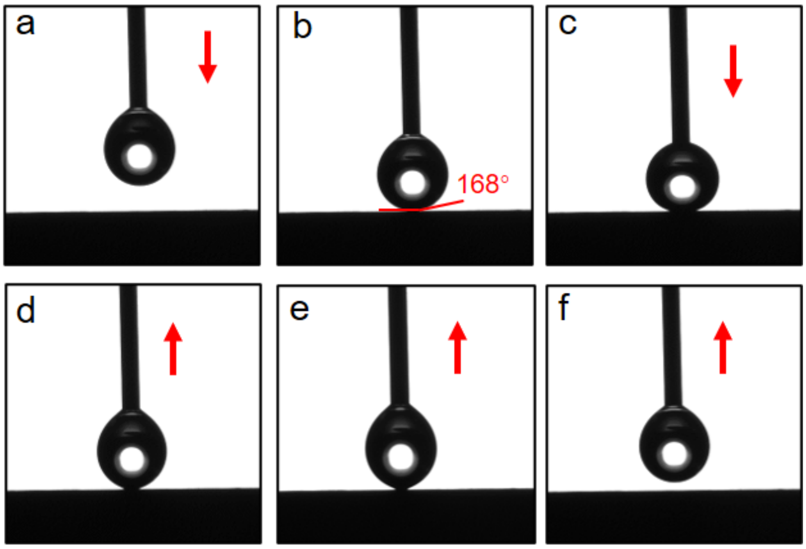

Supplementary Figure 12. Water contact angle dynamic process of ceramic-carbon Janus membranes.

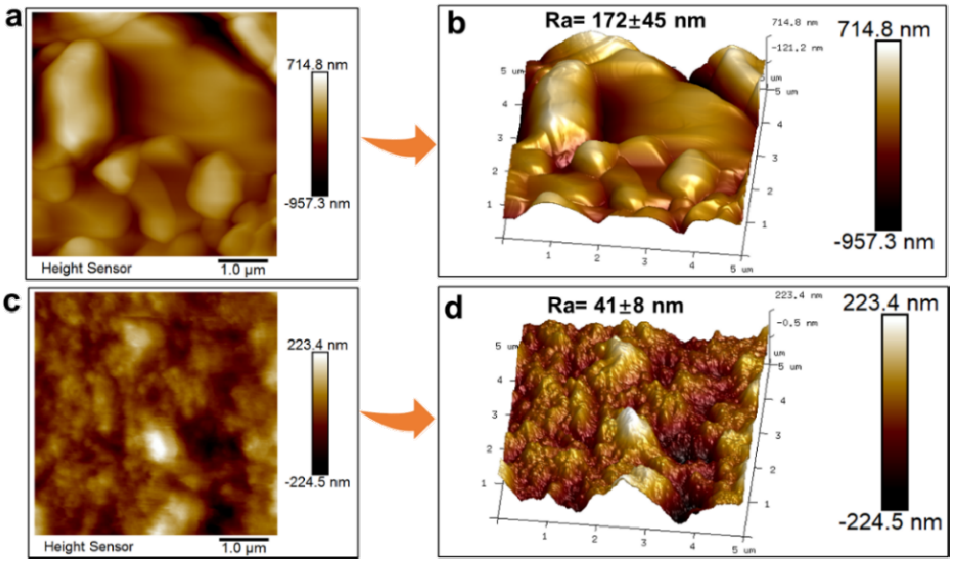

Supplementary Figure 13. Surface AFM images of ceramic membrane and ceramic-carbon Janus membrane: (a) two-dimensional AFM image of ceramic membrane, (a) three-dimensional AFM image with Ra (average roughness) of ceramic membrane, (c) two-dimensional AFM image of ceramic-carbon Janus membrane and (d) three-dimensional AFM image with Ra (average roughness) of ceramic-carbon Janus membrane. The ceramic-carbon Janus membranes show lower surface roughness than aluminum ceramic membranes.

S3 Supplementary Results: Enhanced Solar Absorption and Solar-thermal Effect

Supplementary Table 3. Comparison of the solar absorbance between the ceramic-carbon Janus membranes designed in this work and reported solar-thermal distillation membranes.

Membrane	Solar-thermal material	Solar absorbance (%)	Refs.
PTFE	**PDA-rGO**	84.9	2
PVDF	**MXene**	93.6	3
PVDF-HFP	**Fe_3O_4**	96.0	4
PVDF	**PDA-CNT**	~97.0*	5
PVDF	**TiN**	97.4	6
PVDF	**CB**	97.7	7
PVDF	**cESM-CNT**	99.0	8
Janus membrane	**CNT**	98.1	This work

Notes: PTFE: polytetrafluoroethylene, PVDF: polyvinylidene fluoride, PVDF-HFP: poly (vinylidene fluoride-co-hexafluoropropylene), Al_2O_3: alumina, PDA-rGO: polydopamine-reduced graphene oxide, Fe_3O_4: ferroferric oxide, PDA-CNT: polydopamine-carbon nanotube, TiN: titanium nitride, CB: carbon black, cESM-CNT: carbonized eggshell membrane-carbon nanotube, CNT: carbon nanotube. *Estimated values based on the data presented in the literature.

Supplementary Table 4. Comparison of the thermal conductivity between the ceramic-carbon Janus membranes fabricated in this work and reported ceramic membranes.

**Membrane material**	**Membrane structure**	**Thermal conductivity ($W\ m^{-1}\ K^{-1}$)**	**Refs.**
ZrO_2	Homogeneous pore structure	2.100	[9]
Al_2O_3	Homogeneous pore structure	~4.600*	[10]
β-Sialon	Multi-level pore structure	~1.250*	[10]
γ-$Y_2Si_2O_7$	Homogeneous pore structure	0.497	[11]
α-Si_3N_4	Homogeneous pore structure	0.400	[12]
Janus membrane	Multi-level pore structure	0.447	This work

Notes: ZrO_2: zirconia, Al_2O_3: alumina, γ-$Y_2Si_2O_7$: yttrium silicate, α-Si_3N_4: α-silicon nitride, Janus membranes: alumina-carbon nanotube. *Estimated values based on the data presented in the literature.

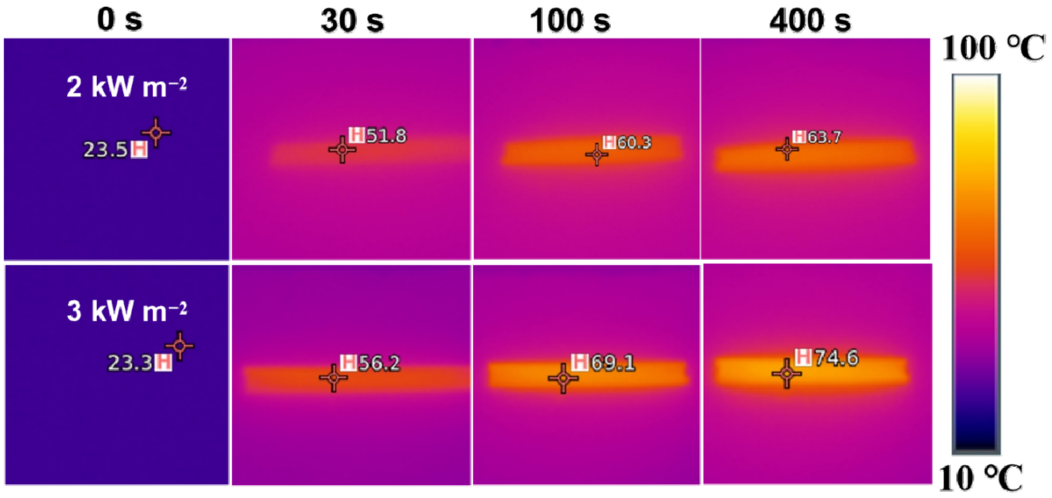

Supplementary Figure 14. Infrared thermal images of ceramic-carbon Janus membranes before (0 s) and after simulated solar light irradiation (2 and 3 kW m^{-2}) for different times (30 s, 100 s, 400 s).

S4. Supplementary Results: Solar-thermal Desalination Performance

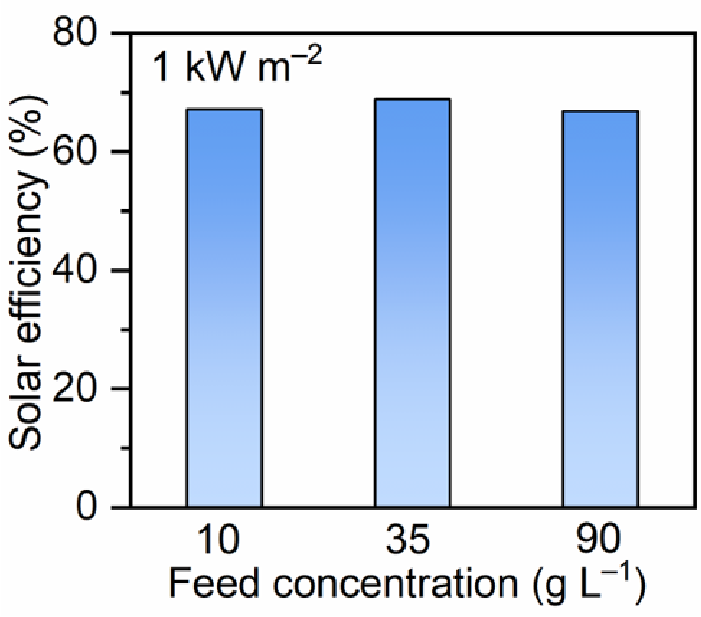

Supplementary Figure 15. Solar conversion efficiency of ceramic-carbon Janus membranes for treatment of saline waters with different salt concentrations (10–90 g L^{-1}) under simulated solar irradiation (1 kW m^{-2}).

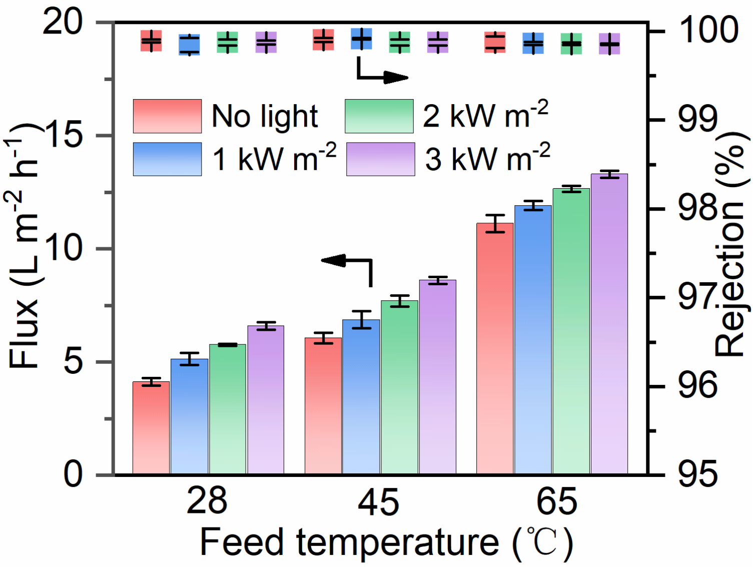

Supplementary Figure 16. Desalination performance (water flux and salt rejection) of ceramic-carbon Janus membranes for treatment of saline waters with different feed temperatures under simulated solar irradiation with different power densities (0–3 kW m^{-2}).

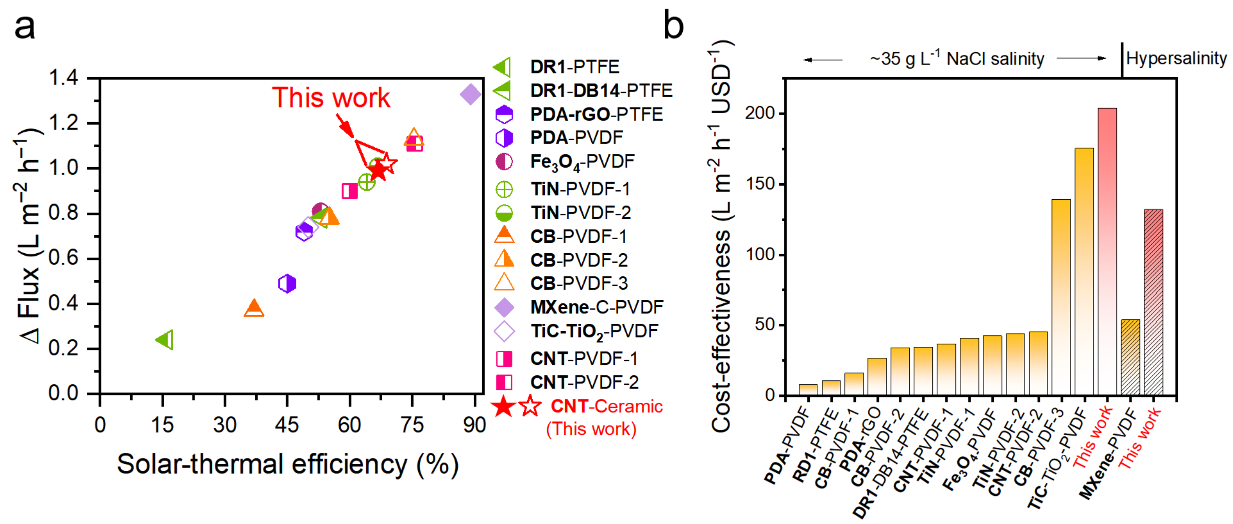

Supplementary Figure 17. Performance and cost-effectiveness comparison between existing state-of-the-art solar-thermal polymeric distillation membranes reported in the literature and the ceramic-carbon Janus membrane (i.e., CNT-Ceramic) fabricated in this work: (a) Δ flux (i.e., the difference of water flux with and without solar illumination) and solar-thermal conversion efficiency, (b) cost-effectiveness (solid pentagram, line-filled bar: 90 g L^{-1} NaCl, hollow pentagram, bar: 35 g L^{-1} NaCl) (Supplementary Table 5).

The techno-economic analysis of desalination technique is important for potential practical applications. To this end, the cost-effectiveness of membrane desalination was defined and then calculated, which reflects the water flux performance per unit mass membrane cost with a unit of L m^{-2} h^{-1} USD^{-1}. This indicator can represent the economics of desalination techniques more accurately. Compared to the state-of-the-art solar-thermal polymeric distillation membranes, our Janus ceramic-carbon membranes exhibit not only higher water flux, but also a higher cost-effectiveness when treating brines with similar salt concentration (Supplementary Figure 18, Supplementary Table 5). In addition, the cost can be further reduced in large-scale massive production of membranes. Both inexpensive membrane materials and reproducable fabrication processes of this Janus membrane would be positively beneficial to future large-scale application and commercialization.

Supplementary Table 5. Comparison of solar-thermal desalination performance between ceramic-carbon Janus membrane designed in this work and reported solar-thermal desalination membranes.

Membrane	Photothermal material	Feed	Temperature (°C)	Photothermal efficiency (%)	Solar density (kW m^{-2})	Δ Flux (L m^{-2} h^{-1})	Flux (L m^{-2} h^{-1})	Cost-effectiveness (L m^{-2} h^{-1} USD^{-1})	Rejection (%)	Refs.
PVDF-2	CNT	35 g L^{-1} NaCl	N.A.	75.6	1	1.11	1.11	45.15	99.8	[8]
PVDF	Fe_3O_4	35 g L^{-1} NaCl	25	53.0	1	0.81	0.97	42.29	99.9	[4]
PVDF-1	TiN	35 g L^{-1} NaCl	20	64.1	1	0.94	0.94	40.86	99.6	[13]
PVDF-1	CNT	35 g L^{-1} NaCl	25	60.0	1	0.90	0.90	36.61	99.9	[14]
PVDF-2	TiN	35 g L^{-1} NaCl	23.3	66.7	1	1.01	1.01	43.90	99.9	[6]
PTFE	PDA-rGO	40 g L^{-1} NaCl	N.A.	49.0	1	0.72	0.72	26.72	99.9	[2]
PTFE	DR1	35 g L^{-1} NaCl	N.A.	16.0	1	0.24	0.24	10.64	99.8	[15]
PTFE	DR1-DB14	Artificial seawater	26.6	53.0	1	0.78	0.78	34.58	99.9	[16]
PVDF-1	CB	10 g L^{-1} NaCl	25	~37.0	0.7	~0.37	~0.37	16.13	99.5	[17]
PVDF	PDA	29 g L^{-1} NaCl	20	45.0	0.75	0.49	0.49	8.00	99.9	[18]
PVDF-2	CB	Seawater	20	~55.1	1	~0.78	0.78	34.00	99.6	[7]
PVDF-3	CB	35 g L^{-1} NaCl	35	75.4	1	1.13	3.19	139.07	99.9	[19]
PVDF	TiC-TiO_2	30 g L^{-1} NaCl	30	~50.0	1	~0.74	~4.18	175.44	99.95	[20]
Janus	CNT	35 g L^{-1} NaCl	28	68.8	1	1.02	5.14	203.79	99.9	This work
C-PVDF	MXene	100 g L^{-1} NaCl	30	89.0	1	1.33	2.88	53.86	99.9	[3]
Janus	CNT	90 g L^{-1} NaCl	28	66.8	1	0.99	3.33	132.03	99.9	This work

Notes: PVDF: polyvinylidene fluoride, PTFE: polytetrafluoroethylene, CNT: carbon nanotube, Fe_3O_4: ferroferric oxide, TiN: titanium nitride, PDA-rGO: polydopamine-reduced graphene oxide, DR1: disperse red 1, DR1-DB14: disperse red 1-disperse blue 14, CB: carbon black. Artificial seawater were prepared from NaCl, $CaCl_2 \cdot 2H_2O$, KCl, KBr, $SrCl_2$, LiCl, $MnCl_2 \cdot 4H_2O$, $AlCl_3 \cdot 6H_2O$ and $Na_2WO_4 \cdot 6H_2O$. N.A.: not available.

S5 Supplementary Methods: Simulation Details and Results

S5.1 Computational Fluid Dynamics Simulation

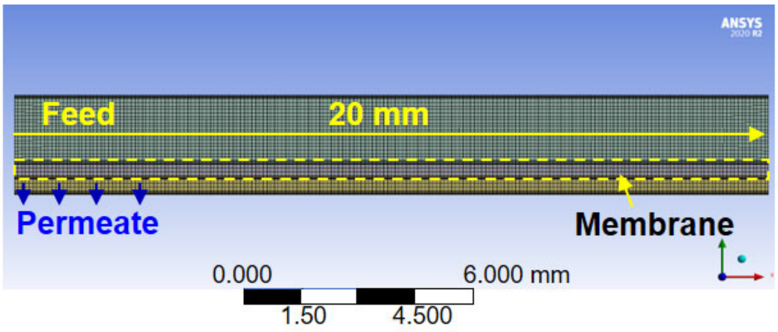

Supplementary Figure 18. Computational domain and mesh grid including three distinct regions: feed (saline water), membrane and permeate (water vapor).

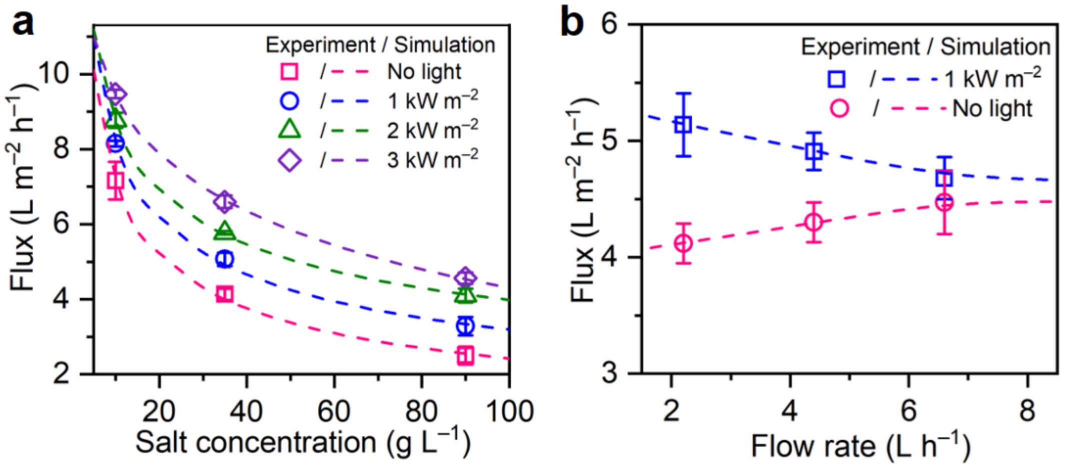

Supplementary Figure 19. Experimental and simulated water fluxes as a function of (a) salt concentration and (b) flow rate.

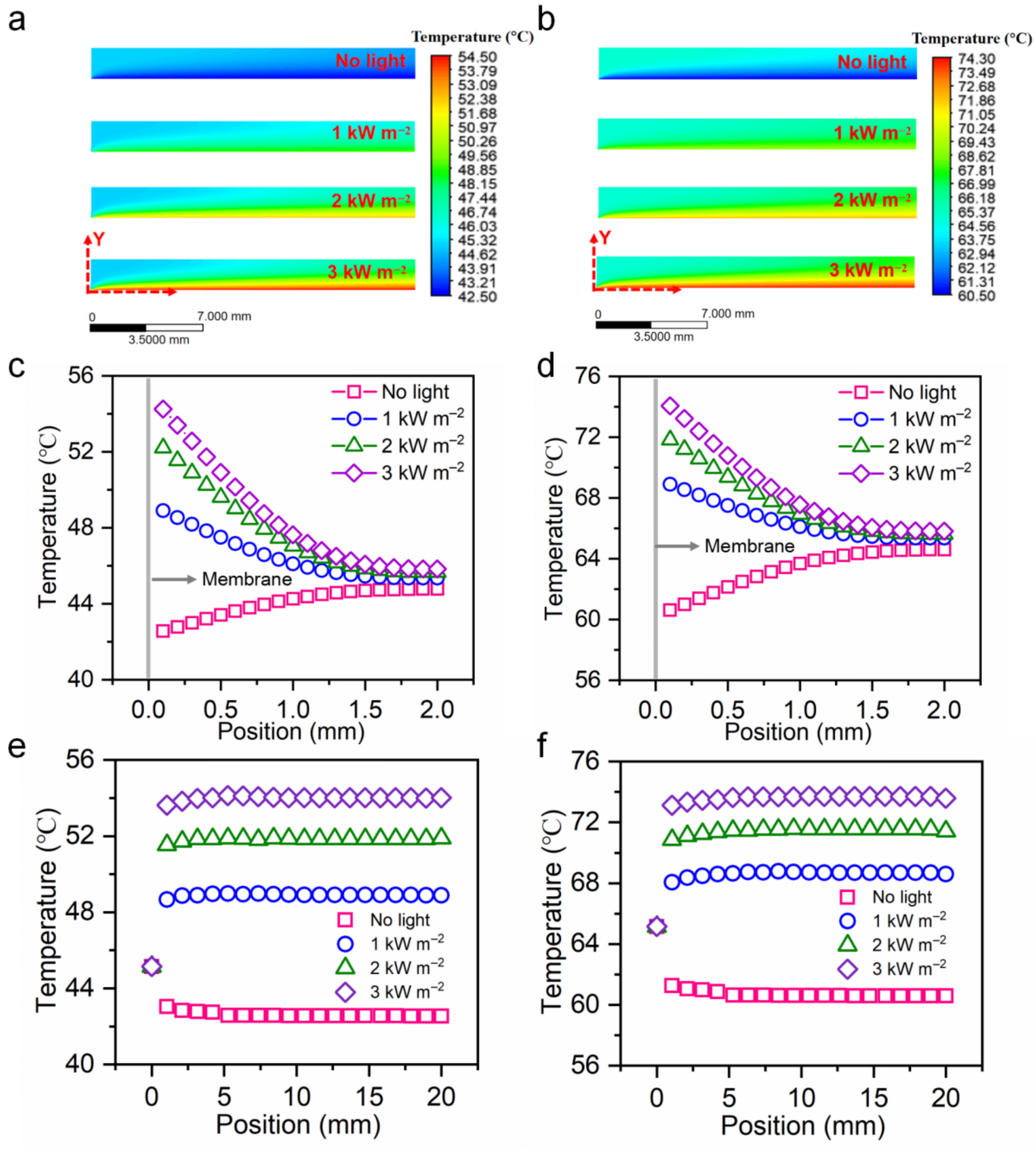

Supplementary Figure 20. Temperature profiles and distribution along different directions at different temperatures: (a, b) the simulated temperature profiles and (c, d) temperature distribution along the direction perpendicular to the membrane surface (i.e., Y direction) under different solar power densities at (a, c) 45 °C and (b, d) 65 °C (0–3 kW m^{-2}). (e, f) The simulated temperature distribution along the membrane direction (i.e., X direction) under different solar power densities at (e) 45 °C and (f) 65 °C (0–3 kW m^{-2}).

S5.2 Mass Transfer Behavior

The water flux of a membrane depends on the vapor pressure difference across the membrane and the mass transfer coefficient of the membrane, which can be given as:

$$J_W = B_M\,(P_{wvf} - P_{wvp}) \tag{2}$$

where J_w (kg m^{-2} s^{-1}) is the water flux, B_M is the mass transfer coefficient, P_{wvf} and P_{wvp} (Pa) are the vapor pressures at membrane interface in feed and permeate, respectively, which can be calculated as follows:

$$P_{wvf} = (1 - 0.5x_{NaCl} - 10x_{NaCl}{}^2) \times X_{wf} \times \exp(23.1964 - (\frac{3816.44}{T_{mf} - 46.13})) \tag{3}$$

$$P_{wvp} = \exp(23.1964 - (\frac{3816.44}{T_{mp} - 46.13})) - 10000 \tag{4}$$

where X_{NaCl} is the mole fraction of NaCl in the feed, X_{wf} is the mole fraction of water. T_{mf} (K) and T_{mp} (K) are the feed-membrane interfacial temperature and permeate-membrane interfacial temperature.

The mass transfer coefficient (B_M) is determined by the Knudsen number and the type of mass transfer. Based on the gas transport model, the mass transfer mechanism of a gas can be categorized into: (1) Knudsen diffusion (K_n >1), (2) molecular diffusion, viscous flow and direct ballistic transport (K_n < 0.01) and (3) a combination of diffusion Knudsen and viscous flow (0.01 < K_n < 1), which can be expressed by the following equation.

$$K_n = \frac{K_B T_m}{d_{pore}\sqrt{2}\pi P_m \sigma^2} \tag{5}$$

$$K_n > 1 \quad B_M = B_K = (\frac{2\varepsilon r}{3\delta\tau}) \times (\frac{8MW_w}{\pi R T_m})^{0.5} \tag{6}$$

$$K_n < 1 \quad B_M = B_m = \frac{MW_w \varepsilon PD}{RT_m \delta\tau P_g} \tag{7}$$

$$0.01 < K_n < 1 \qquad B_M = (\frac{1}{B_K} + \frac{1}{B_m})^{-1} \tag{8}$$

where λ (m) is the mean free path, d_{pore} (m) is the diameter of the membrane pores, k_B is the Boltzmann constant (1.38×10^{-23} J K^{-1}), T_m (K) is the absolute temperature, P_m (Pa) is the mean pressure within the membrane pores, σ is the diameter of water vapor molecule (2.64×10^{-10} m), MW_w is the molecular weight of water (18 g mol^{-1}), R is the universal gas

constant (8.314 J K^{-1} $mole^{-1}$), P_g (Pa) is the total pressure inside the membrane pores (the sum of the partial pressures of air and liquid), and D is the water diffusion coefficient (D = $1.895 \times 10^{-5}\, T_m^{2.072}/P$)

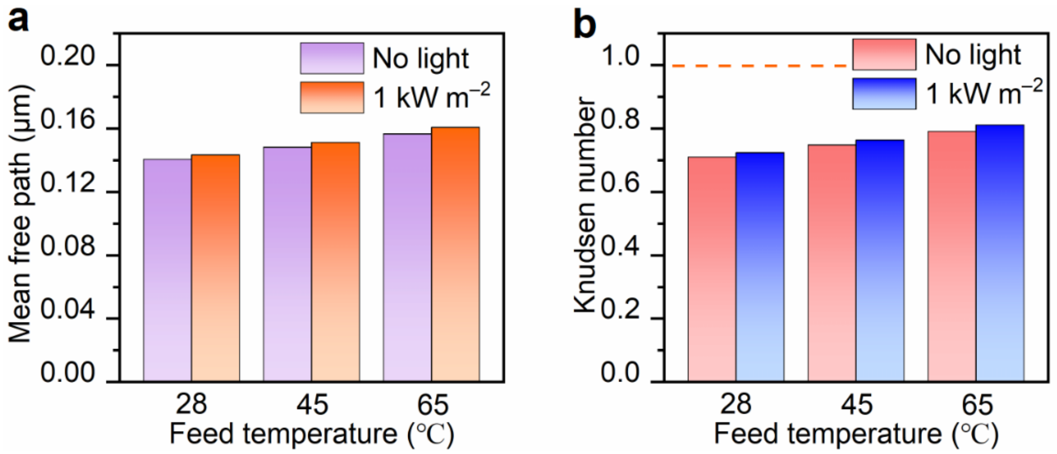

Supplementary Figure 21. Effect of feed temperature on the transport properties of water vapor molecules without and with simulated solar illumination (1 kW m^{-2}): (a) mean free path and (b) Knudsen number.

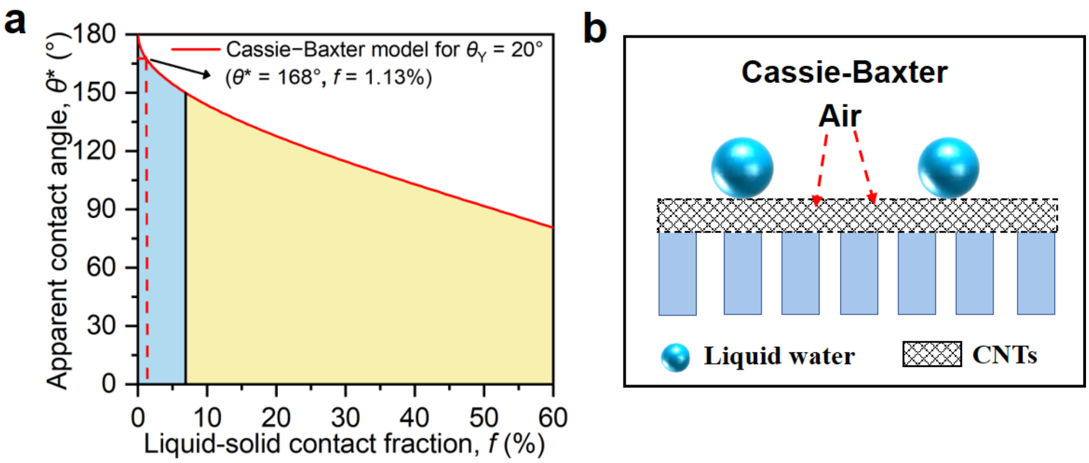

Supplementary Figure 22. Surface wetting properties of the ceramic-carbon Janus membranes: (a) relationship between apparent contact angle θ* and liquid-solid contact fraction f for an ideal Cassie-Baxter state at the Young's contact angle θ_Y (light blue color, superhydrophobic zone; light yellow color, hydrophobic zone) and (b) a schematic illustration of a water droplet in the Cassie-Baxter state on the membrane surface.

S5.3 Molecular Dynamics Simulation

Molecular dynamics simulations were conducted using Materials Studio 2023 (BIOVIA). In addition to water-water intermolecular interaction, the interaction between water molecules and CNT layers was only considered because the ceramic substrate of our Janus membrane did not interact with water molecules due to the superhydrophobic feature of CNT layer surface. Amorphous cell module was used to construct the water layer and CNT layer. Specifically, for water layer, 500 water molecules were packed into a simulation cell with a dimension of 25 Å×25 Å×23 Å at a predefined density of 1.0 g cm^{-3} (Supplementary Figure 23a). In CNT layer, the CNT was constructed with a comparable dimension of 25 Å×25 Å×24.6 Å to water layer (Supplementary Figure 23b). The obtained water and CNT layers were geometrically optimized for 15000 steps using Smart algorithm. To obtain the CNT-water system, the water layer and CNT layer were assembled along the vertical direction with a intergap of 2 Å. Meanwhile, a vacuum slab of 150 Å was added at the upper surface of the water layer to avoid the effects from the periodic boundary. Similarly, two as-prepared water layers were assembled together to construct the water-water (i.e., pure water) system.

After minimizing the energy of water-water (i.e., pure water) and water-CNT systems using geometric optimization, molecular dynamics simulations for 500 ps with a step length of 1 fs were performed under the NVT (constant temperature and volume) ensemble at 313 K and 353 K to investigate the temperature effects on the water evaporation. The intramolecular energy was determined using the Cohesive Energy Density Analysis Tool in Materials Studio using COMPASS III Force field, which incorporates bond stretching, angle bending, torsional interactions, and non-bonded interactions. COMPASS III is one of the universal force fields based on ab initio simulations. The force field for water in COMPASS III is a flexible model incorporating the inter- and intramolecular motion of a water molecule, which is appropriate for investigating the microscopic formation of water molecular structures.[21] The cutoff radius of short-range interactions was set to 1.25 nm, and the long-range electrostatic interactions were calculated by the Ewald method.[22] The charge distribution in a water molecule was set to −0.41 eC for hydrogen and 0.82 eC for oxygen, which were determined using COMPASS III. The carbon atoms constituting MWCNT were charge-neutral.

The hydrogen bond analysis was performed using the Hydrogen Bond Analysis Tool in Materials Studio. A hydrogen bond was considered to exist when the distance between the donor and acceptor is less than 2.5 Å, and the donor-hydrogen-acceptor angle is greater than 90°.

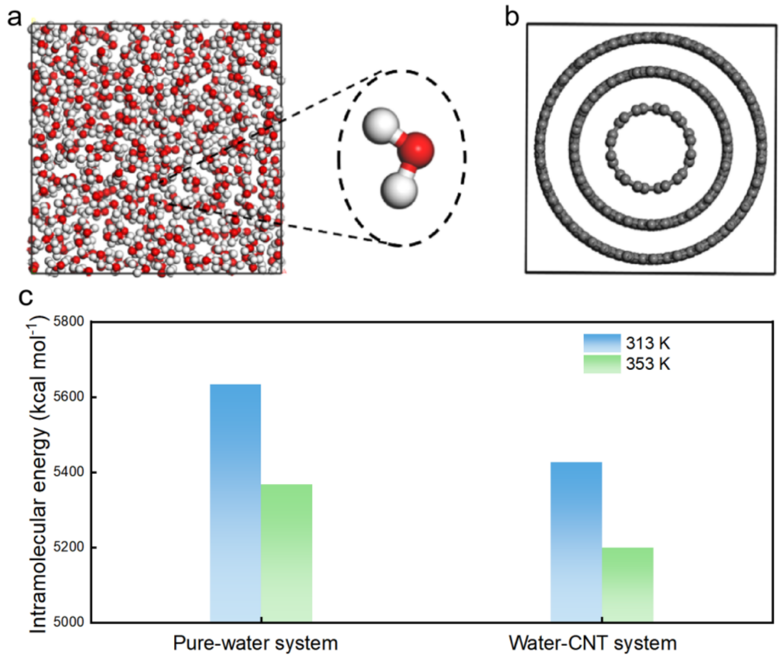

Supplementary Figure 23. Simulation models and intramolecular energy of two systems: (a) simulation model of water layer, (b) simulation model of CNT layer, and (c) intramolecular energy of water layer of the water-CNT and water-water (i.e., pure water) systems.

S6 Supplementary Results: Solar-thermal Desalination of Challenging Saline Waters

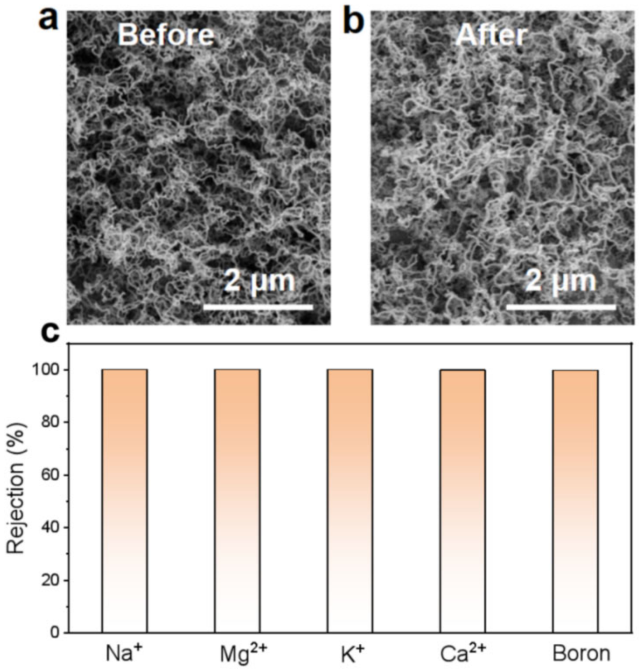

Supplementary Figure 24. Surface FE-SEM images and rejection of ceramic-carbon Janus membranes for treatment of seawater: surface FE-SEM image (a) before and (b) after solar-thermal desalination operation, and (c) rejection of five primary species.

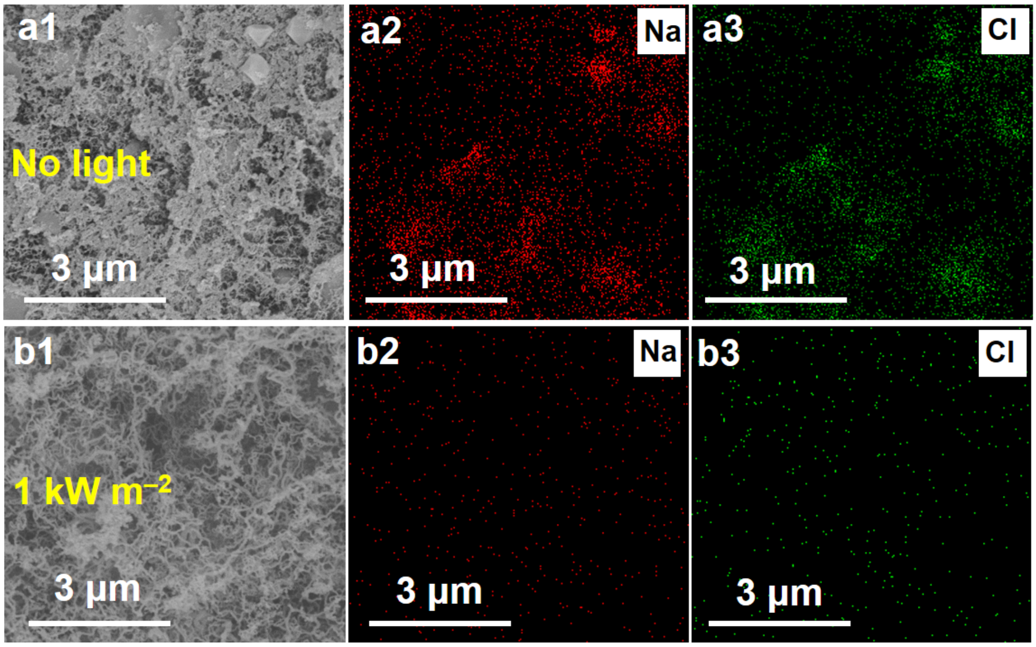

Supplementary Figure 25. SEM-EDS images of ceramic-carbon Janus membranes after operation for treatment of hypersaline water (90 g L^{-1} NaCl) (a1–a3) without and (b1–b3) with solar illumination (1 kW m^{-2}).

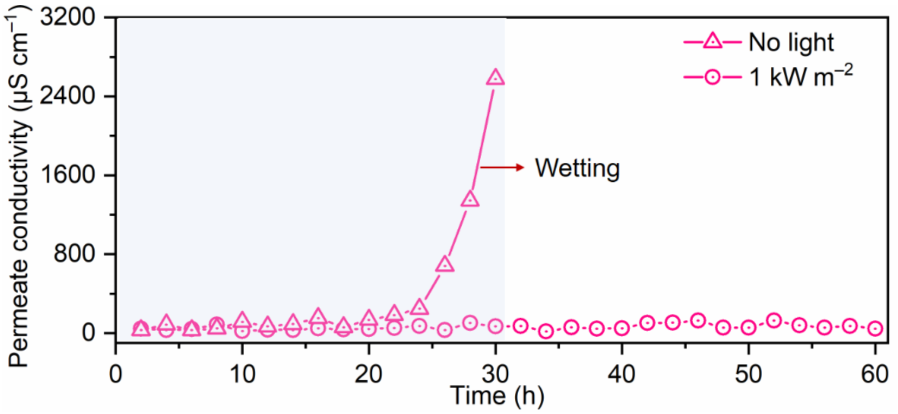

Supplementary Figure 26. Permeate conductivity of ceramic-carbon Janus membranes for treatment of hypersaline water (90 g L^{-1} NaCl) with presence of $CaSO_4$ (3 g L^{-1}) without and with solar illumination (1 kW m^{-2}).

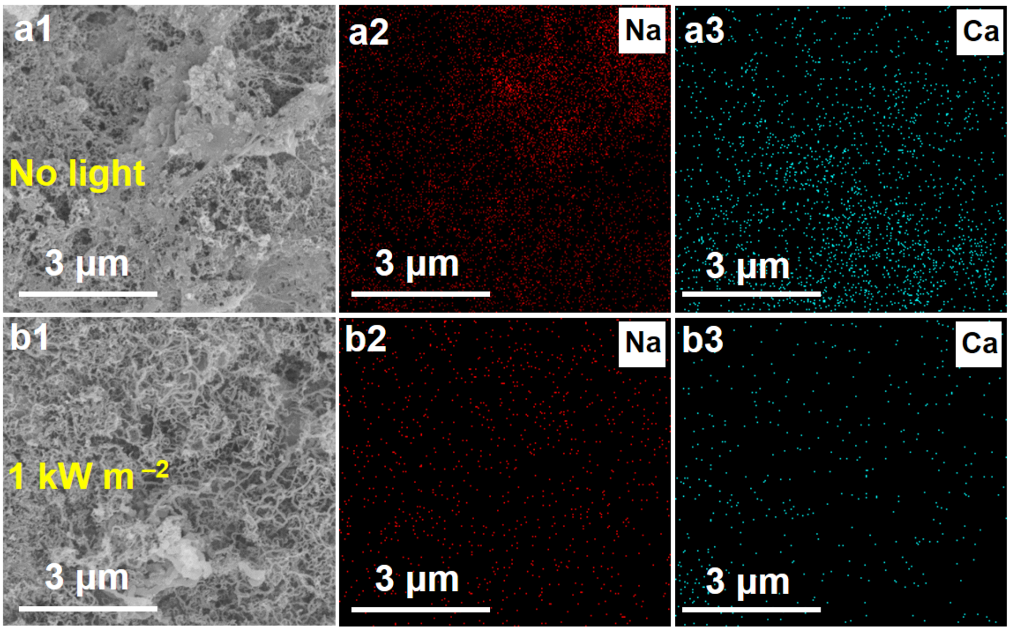

Supplementary Figure 27. SEM-EDS images of ceramic-carbon Janus membranes after operation for treatment of hypersaline waters containing inorganic gypsum (90 g L^{-1} NaCl and 3 g L^{-1} $CaSO_4$) (a1–a3) without and (b1–b3) with solar illumination (1 kW m^{-2}).

S7 Supplementary Results: Design and Solar-thermal Desalination Performance of Flat Membranes

The design strategy of solar-thermally enhanced tubular Janus membranes can also be extended to the fabrication of flat Janus membranes. Large dimension alumina flat ceramic membranes were specially designed and fabricated via the combined technique of phase inversion tape casting and high-temperature sintering (Supplementary Figure 28a).[23] The ceramic membranes show a special structure featuring a ultralong finger-like layer with vertical macro-pores. They have much high water permeance, outperforming commercial flat-sheet ceramic membranes. In situ construction of CNT onto flat ceramic membranes was then carried out via chemical vapor deposition (CVD). The fabricated ceramic-carbon Janus flat membranes have an interconnected CNT network structure, featuring superhydrophobic property (water contact angle ~161.4°). They also show strong and broad solar absorption (~97.7%) in the wavelength ranging from 300 nm to 2400 nm (Supplementary Figure 29). After irradiation with a power density of 1 kW m^{-2} and 3 kW m^{-2} for 25 min, the surface temperature of the membranes increased to 50.7 °C and 79.3 °C (Supplementary Figure 29), respectively, demonstrating its promising solar-thermal conversion ability due to the presence of the CNT layer. In addition, solar-thermal desalination performance of the fabricated Janus flat membranes was tested under simulated solar illumination. The results indicate that the solar-thermal Janus flat membranes show a higher water flux with solar illumination than that without solar illumination (Supplementary Figure 29). Therefore, it is believed that Janus membranes fabricated using this design strategy will have a application potential in efficient solar-thermal interfacial desalination.

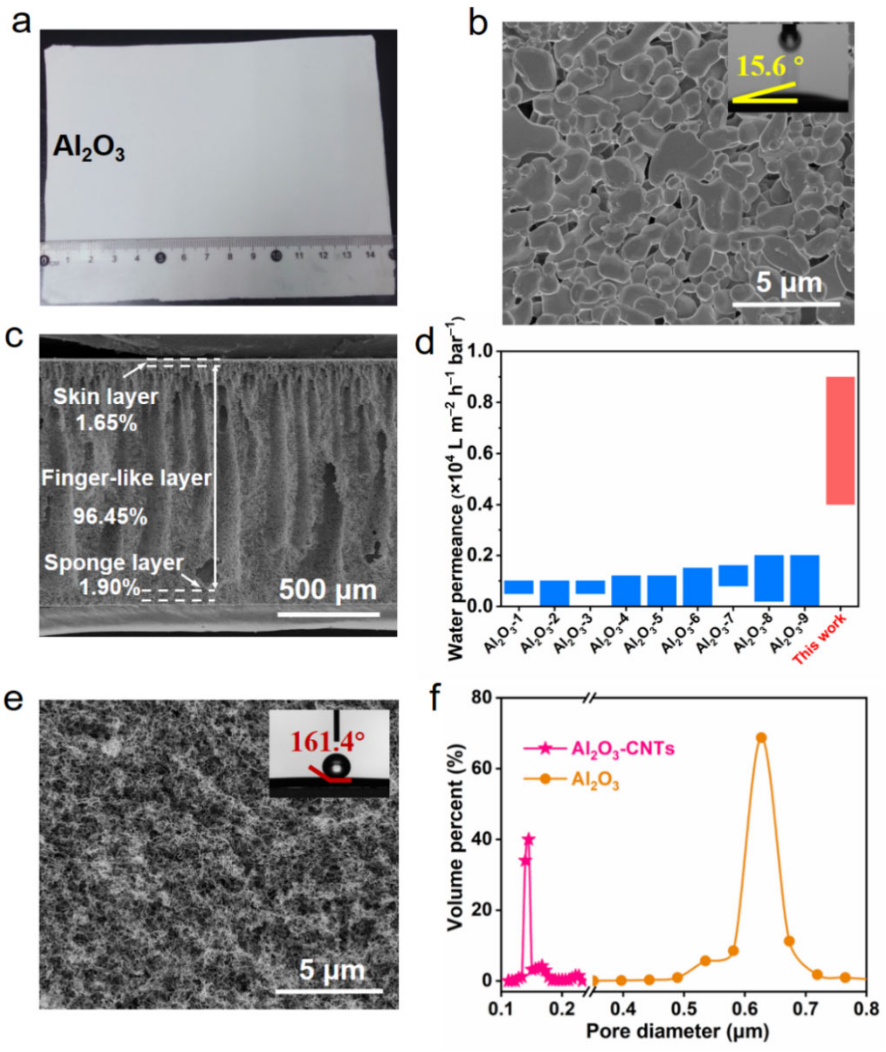

Supplementary Figure 28. Morphology, permeation property and pore size distribution of flat-sheet ceramic membrane and ceramic-carbon Janus membranes. (a) Photograph of flat-sheet Al_2O_3 ceramic membrane green body. (b) Surface and (c) cross-section SEM images of flat-sheet Al_2O_3 membranes (the inset of Supplementary Figure 28b shows water contact angle measured at room temperature (~25 °C) on the Al_2O_3 membrane surface). (d) Comparison of water permeance between flat-sheet Al_2O_3 membrane in this work and other commercial membranes. (e) Surface SEM image of flat-sheet ceramic-carbon Janus membranes (the inset of Supplementary Figure 28b shows water contact angle measured at room temperature (~25 °C) on the membrane surface). (f) Pore size distribution of flat-sheet Al_2O_3 and ceramic-carbon Janus membranes (Al_2O_3-CNT).

Supplementary Table 6. Comparison of the parameters between commercial ceramic membranes and ceramic membrane designed in this work.

**Membrane**	**Configuration**	**Pore Size (μm)**	**Water Permeance ($L\ m^{-2}\ h^{-1}\ bar^{-1}$)**	**Supplier**	**Website**
Al_2O_3-1	Tubular	0.1-1.0	500-1000	Shanghai Silicon Garden Membrane Technology Co., Ltd.	https://www.sicermem.com/
Al_2O_3-2	Tubular	0.1	1000	Zibo Dongqiang Membrane Technology Co., Ltd.	http://www.zbdongqiang.cn/
Al_2O_3-3	Flat-sheet	0.1-0.4	500-1000	Uniaxial Dry Pressing	
Al_2O_3-4	Tubular	0.1	1200	Yaan Walklin Environmental Technology Co., Ltd.	https://www.weclean-tech.com/
Al_2O_3-5	Tubular	0.1-0.2	1200	Jiangsu Lvdun Membrane Technology Co., Ltd.	https://www.ledonmo.com/
Al_2O_3-6	Flat-sheet	0.13-0.15	1500	Shandong Industrial Ceramics Research & Design Institute Co., Ltd.	http://www.sinofinecera.cn/
Al_2O_3-7	Tubular	0.02-0.1	800-1600	Zibo Yuding New Material Technology Co., Ltd.	http://yudingxincai.qinghuan.com/
Al_2O_3-8	Tubular	0.2-2.0	200-2000	Shanghai Keyu Water Treatment Technology Co., Ltd.	http://www.chemarea.com/
Al_2O_3-9	Tubular	0.1	2000	Jiangsu Yixing Non-metallic Chemical Machinery Plant Co., Ltd.	http://www.yxhjc.com/
Al_2O_3	Flat-sheet	0.4-0.9	4000-9000	This work	

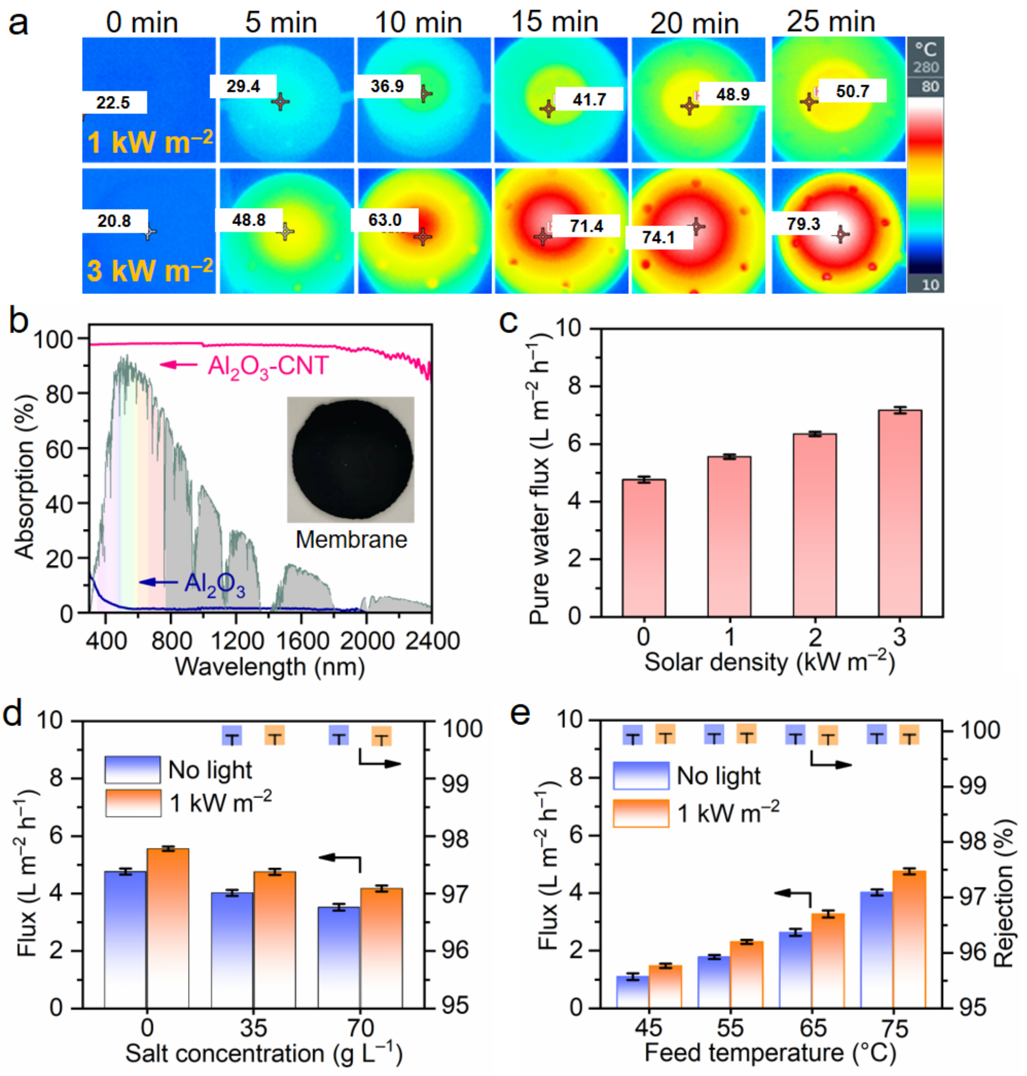

Supplementary Figure 29. Solar-thermal properties and desalination performance of flat-sheet ceramic-carbon Janus membrane. (a) Infrared thermal images of ceramic-carbon Janus membrane before and after simulated solar light irradiation (1, 3 kW m^{-2}) for different times. (b) The UV vis–NIR absorption spectra of aluminum ceramic membrane and ceramic-carbon Janus membrane in the wavelength ranging from 300 nm to 2400 nm (The inset shows the photograph of flat-sheet ceramic-carbon Janus membrane). (c) Pure water flux of ceramic-carbon Janus membrane at different solar densities (0–3 kW m^{-2}) at 75 °C. Comparison of desalination performance (water flux and salt rejection) of flat-sheet ceramic-carbon Janus membrane for treatment of saline waters with (d) different feed concentrations and (e) feed temperatures under simulated solar irradiation with different power densities (0–1 kW m^{-2}).

Supplementary References

1. Zhu, L.; Dong, X.; Xu, M.; Yang, F.; Guiver, M. D.; Dong, Y., Fabrication of mullite ceramic-supported carbon nanotube composite membranes with enhanced performance in direct separation of high-temperature emulsified oil droplets. Journal of Membrane Science 2019, 582, 140-150.
2. Huang, L.; Pei, J.; Jiang, H.; Hu, X., Water desalination under one sun using graphene-based material modified PTFE membrane. Desalination 2018, 442, 1-7.
3. Zhang, B.; Wong, P. W.; Guo, J.; Zhou, Y.; Wang, Y.; Sun, J.; Jiang, M.; Wang, Z.; An, A. K., Transforming Ti3C2Tx MXene's intrinsic hydrophilicity into superhydrophobicity for efficient photothermal membrane desalination. Nature Communications 2022, 13, (1), 1-10.
4. Li, W.; Chen, Y.; Yao, L.; Ren, X.; Li, Y.; Deng, L., Fe3O4/PVDF-HFP photothermal membrane with in-situ heating for sustainable, stable and efficient pilot-scale solar-driven membrane distillation. Desalination 2020, 478, 114288.
5. Wang, Y.; Liao, X.; Zhang, X.; Shi, M.; You, X.; Liao, Y.; Razaqpur, A. G., Engineering Surface Wettability to Alleviate Membrane Scaling in Photothermal Membrane Distillation. ACS ES&T Water 2022, 3, (7), 1847-1854.
6. Farid, M. U.; Kharraz, J. A.; An, A. K., Plasmonic titanium nitride nano-enabled membranes with high structural stability for efficient photothermal desalination. ACS Applied Materials & Interfaces 2021, 13, (3), 3805-3815.
7. Gong, B.; Yang, H.; Wu, S.; Yan, J.; Cen, K.; Bo, Z.; Ostrikov, K. K., Superstructure-enabled anti-fouling membrane for efficient photothermal distillation. ACS Sustainable Chemistry & Engineering 2019, 7, (24), 20151-20158.
8. Han, X.; Wang, W.; Zuo, K.; Chen, L.; Yuan, L.; Liang, J.; Li, Q.; Ajayan, P. M.; Zhao, Y.; Lou, J., Bio-derived ultrathin membrane for solar driven water purification. Nano Energy 2019, 60, 567-575.
9. Arumugham, T.; Kaleekkal, N. J.; Gopal, S.; Nambikkattu, J.; Rambabu, K.; Aboulella, A. M.; Wickramasinghe, S. R.; Banat, F., Recent developments in porous ceramic membranes for wastewater treatment and desalination: A review. Journal of Environmental Management 2021, 293, 112925.
10. Wang, J.-W.; Li, L.; Zhang, J.-W.; Xu, X.; Chen, C.-S., β-Sialon ceramic hollow fiber membranes with high strength and low thermal conductivity for membrane distillation. Journal of the European Ceramic Society 2016, 36, (1), 59-65.

11. Yang, M.-Y.; Wang, J.-W.; Li, L.; Dong, B.-B.; Xin, X.; Agathopoulos, S., Fabrication of low thermal conductivity yttrium silicate ceramic flat membrane for membrane distillation. Journal of the European Ceramic Society 2019, 39, (2-3), 442-448.

12. Li, L.; Wang, J.-W.; Zhong, H.; Hao, L.-Y.; Abadikhah, H.; Xu, X.; Chen, C.-S.; Agathopoulos, S., Novel α-Si3N4 planar nanowire superhydrophobic membrane prepared through in-situ nitridation of silicon for membrane distillation. Journal of Membrane Science 2017, 543, 98-105.

13. Zhang, Y.; Li, K.; Liu, L.; Wang, K.; Xiang, J.; Hou, D.; Wang, J., Titanium nitride nanoparticle embedded membrane for photothermal membrane distillation. Chemosphere 2020, 256, 127053.

14. Huang, J.; Hu, Y.; Bai, Y.; He, Y.; Zhu, J., Novel solar membrane distillation enabled by a PDMS/CNT/PVDF membrane with localized heating. Desalination 2020, 489, 114529.

15. Fujiwara, M., Water desalination using visible light by disperse red 1 modified PTFE membrane. Desalination 2017, 404, 79-86.

16. Fujiwara, M.; Kikuchi, M., Solar desalination of seawater using double-dye-modified PTFE membrane. Water Research 2017, 127, 96-103.

17. Dongare, P. D.; Alabastri, A.; Pedersen, S.; Zodrow, K. R.; Hogan, N. J.; Neumann, O.; Wu, J.; Wang, T.; Deshmukh, A.; Elimelech, M., Nanophotonics-enabled solar membrane distillation for off-grid water purification. Proceedings of the National Academy of Sciences 2017, 114, (27), 6936-6941.

18. Wu, X.; Jiang, Q.; Ghim, D.; Singamaneni, S.; Jun, Y.-S., Localized heating with a photothermal polydopamine coating facilitates a novel membrane distillation process. Journal of Materials Chemistry A 2018, 6, (39), 18799-18807.

19. Chen, Y.-R.; Xin, R.; Huang, X.; Zuo, K.; Tung, K.-L.; Li, Q., Wetting-resistant photothermal nanocomposite membranes for direct solar membrane distillation. Journal of Membrane Science 2021, 620, 118913.

20. Liu, J.; Guo, H.; Sun, Z.; Li, B., Preparation of photothermal membrane for vacuum membrane distillation with excellent anti-fouling ability through surface spraying. Journal of Membrane Science 2021, 634, 119434.

21. Maekawa, Y.; Sasaoka, K.; Yamamoto, T., Structure of water clusters on graphene: A classical molecular dynamics approach. Japanese Journal of Applied Physics 2018, 57, (3), 035102.

22. Ewald, P. P., Die Berechnung optischer und elektrostatischer Gitterpotentiale. Annalen der Physik

1921, 369, (3), 253-287.

23. Miao, M.; Liu, T.; Bai, J.; Wang, Y., Engineering the wetting behavior of ceramic membrane by carbon nanotubes via a chemical vapor deposition technique. Journal of Membrane Science 2022, 648, 120357.
